# Diffuse Idiopathic Skeletal Hyperostosis Involving Cervical and Lumbar Spine Presenting with Dysphagia: A Case Report

**Published:** 2017-07

**Authors:** Ramanuj Sinha, Neeraj Aggarwal, Sirshak Dutta, Avijit Choudhury, Sanjoy-Kumar Ghosh, Debasis Guha

**Affiliations:** 1 *Department of Otorhinolaryngology, Medical College, Kolkata, India.*; 2 *Senior Divisional Medical Officer, B R Singh Hospital (Eastern Railways), Kolkata, India*

**Keywords:** Diffuse Idiopathic Skeletal Hyperostosis, Dysphagia, Forestier disease, Cervical spine, Lumbar spine

## Abstract

**Introduction:**

Diffuse Idiopathic Skeletal Hyperostosis (DISH) is a very rare cause of dysphagia when it occurs in the cervical spine. It can also affect the lumbar region where it causes deformity.

**Case Report:**

In this article, a rare case of Diffuse Idiopathic Skeletal Hyperostosis involving both the cervical and lumbar spine, presenting with dysphagia and spinal stiffness leading to a stooping posture, is reported.

**Conclusion:**

Cases of simultaneous involvement of cervical and lumbar vertebrae by Diffuse Idiopathic Skeletal Hyperostosis, presenting with symptoms of both area involvement, are rarely reported in the English literature. When investigating a case of dysphagia, a high level of suspicion is required to diagnose such a condition.

## Case Report

**Fig 1:**
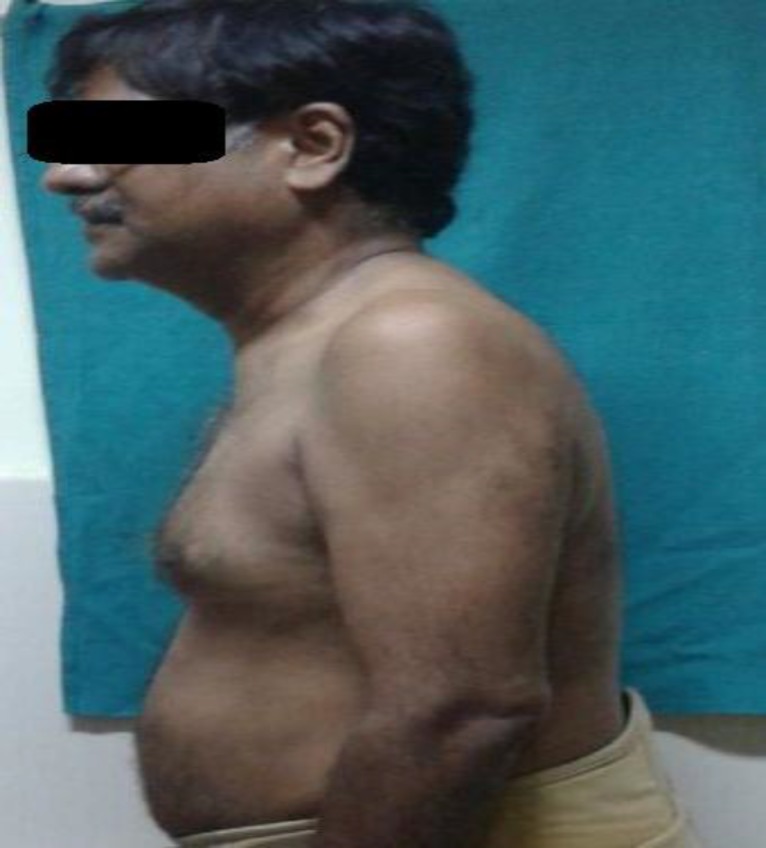
Typical posture of the patient

A 49-year-old gentleman came to our institute with dysphagia for the last two years. The dysphagia was gradually progressive in nature and was more for solid than liquid. There was no associated pain during deglutition. The patient had been treated by several physicians for one and a half years for the same complaint but without any relief. The patient lost 5-6kg of weight during this period. He was earlier diagnosed with cervical spondylosis and treated with a cervical collar, analgesics, and neck exercises. However, nothing could relieve his main complaint of dysphagia. When the patient arrived in our clinic, his body posture was stooping forward and he was having difficulty in straightening his torso over his pelvis (Fig.1).

**Fig 2:**
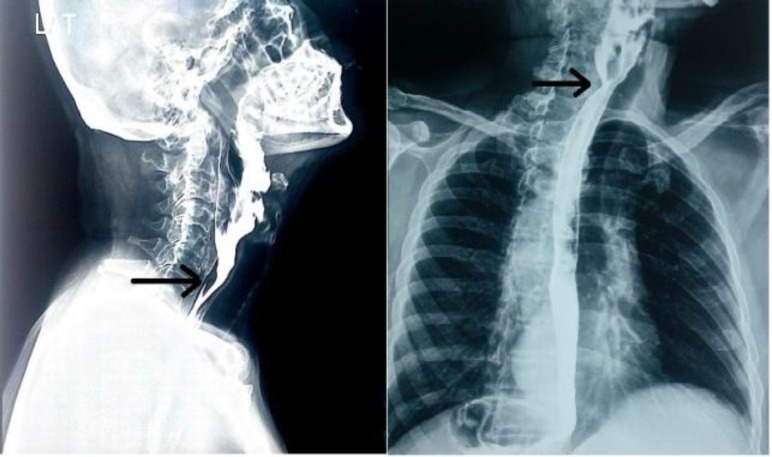
Ossification seen in the anterolateral aspect of C3-T1 vertebrae

The patient was a known hypertensive and was prescribed tablet telmisartan 40mg since 5 years. The patient was also on metformin 500 mg twice daily for type II diabetes mellitus.During a detailed clinical examination, there was no obvious pathology found in the oraphaynx and laryngopharynx. No neck nodes were clinically palpable. All other systemic examinations were not significant. All the routine blood investigations were normal. Barium swallow oesophagus was suggested and it showed a narrowing of the lumen in the hypopharynx and cervical part of the oesophagus. Ossification was seen in the anterolateral aspect of the C3-T1 vertebrae (Fig.2). 

**Fig 3:**
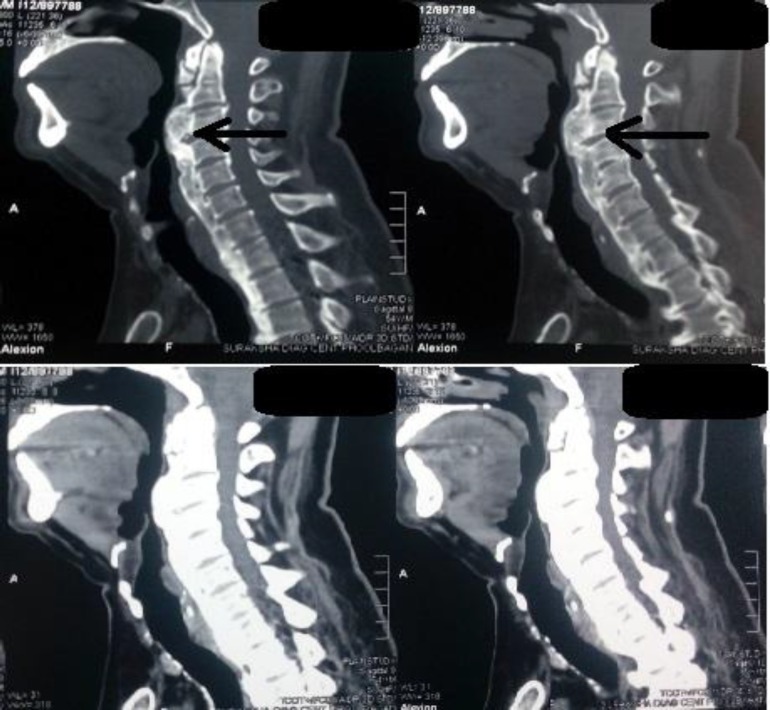
Narrowing of lumen by ossification

The rest of the oesophagus was normal. This was confirmed by computed tomography of the vertebral column showing syndesmophytes and anterior longitudinal ligament ossification at the cervico-dorsal spine. This was impinging upon the posterior pharyngeal wall and oesophagus confirming the diagnosis of Diffuse Idiopathic Skeletal Hyperostosis (DISH) (Fig.3). 

**Fig 4:**
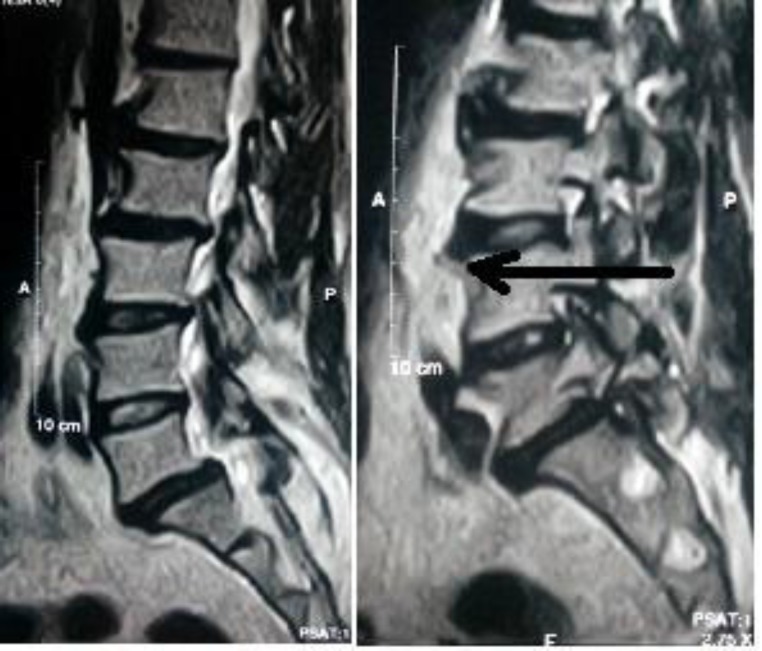
Ossification of the lumbar spine from L1-L5 vertebrae

Upper Gastro-intestinal tract endoscopy, another important investigation for dysphagia, was not performed in this case as there was no intraluminal mass lesion detected in the CT scan. There was another segment showing ossification of the lumbar spine from L1-L5 vertebrae (Fig.4). We advised the patient to have a surgical removal of the osteophytes; but he refused to do any sort of surgical intervention. So we advised him to avoid solid food and ingest soft semisolid food in a small bolus instead. After a 3-month of follow up, the patient wasdoing well and was satisfied with his diet therapy. 

## Discussion

Forestier and Rotes-Querol described an ankylosing spondylitis in elderly people and called them Forestier disease (1)**. **Resnick, in 1970, coined the term Diffuse Idiopathic Skeletal Hyperostosis which is characterized by a) ossification of the anterior longitudinal ligament of at least four contiguous vertebral bodies, most commonly those of the thoracic spine. b) minimal degree of degenerative disc disease.c) absence of apophyseal joint ankylosis and sacroiliac erosions (2,3). 

DISH, though common and reported in various orthopedic and rheumatology literature (4), is rarely reported in otorhinolaryngology literature. Holton et al. calculated that the prevalence of DISH is about 40% in a population of more than 65 years of age (5).Most cases of DISH are asymptomatic. Symptomatic patients are generally presented to orthopedicians with neck pain or otolaryngologists with dysphagia (3,6-10). Diffuse idiopathic skeletal hyperostosis (DISH) is mainly a disease of elderly men. As the bony projections in DISH are away from spinal cords, it rarely produces compressive symptoms.

According to a meta-analysis by Dutta et al (11), only 73 cases of Forestier disease presenting with dysphagia were reported till 2010 in the English literature and most of the patients were elderly male in the seventh or eighth decade. Dysphagia was reported as the most common presenting symptom of DISH affecting the cervical spine with a prevalence rate of 17-28% (12).Only a very few cases were reported in patients of less than 50 years of age, proving the rarity of the present case. The diagnosis mainly depends on radiology. Barium swallow oesophagus generally shows a narrow segment due to compression by osteophytes, such as in the present case. Computed tomography confirms the diagnosis by showing ossification of the anterior spinal ligament. The novelty of the present case was the presence of dysphagia with simultaneous involvement of the cervicodorsal spine from C3- T1 and lumbar spine from L1-L5. Co-existence of all those features in a patient is seldom reported. Although the main treatment of DISH is surgery, many patients are relieved with conservative measures in the form of a compensatory strategy and rehabilitative therapy for dysphagia, especially much older patients who are medically unfit for surgery or who refuse surgery.

## Conclusion

Diffuse Idiopathic Skeletal Hyperostosis (DISH) is mainly a disease of older people, but can also occur in middle aged patients. Simultaneous involvement of the cervical and lumbar region of the vertebral column, presenting with stiffness of the spine as well as dysphagia, is rare. The possibility of this condition should be kept in mind while addressing a patient with low back pain, stiffness, and dysphagia. 
